# Spinal Cord Injury in Real Time: Intra-Operative Ultrasound for Acute Phase Examination in Non-Human Primates

**DOI:** 10.3390/brainsci15091005

**Published:** 2025-09-17

**Authors:** Eleni Sinopoulou, Michelle W. Chow, Numaira Obaid, Emily Chong, Yvette S. Nout-Lomas, Rachele Wurr, Ryan Macon, J. Russell Huie, Adam R. Ferguson, Mark H. Tuszynski, Michael S. Beattie, Jacqueline C. Bresnahan, Carolyn J. Sparrey

**Affiliations:** 1 Department of Neurosciences, University of California—San Diego, La Jolla, CA 92093, USA; mwchow@health.ucsd.edu (M.W.C.); eychong@ucsd.edu (E.C.); mtuszynski@health.ucsd.edu (M.H.T.); 2 Mechatronic Systems Engineering, Simon Fraser University, Surrey, BC V3TOA3, Canada; numaira_obaid@sfu.ca; 3 International Collaboration on Repair Discoveries (ICORD), Vancouver, BC V5Z 1M9, Canada; 4 College of Veterinary Medicine and Biomedical Sciences, Colorado State University, Fort Collins, CO 80521, USA; yvette.nout-lomas@colostate.edu; 5 California National Primate Research Center, University of California, Davis, CA 95616, USA; rlwurr@ucdavis.edu (R.W.); rjmacon@ucdavis.edu (R.M.); 6 Department of Neurosurgery, University of California, San Francisco, CA 94143, USA; russell.huie@ucsf.edu (J.R.H.); adam.ferguson@ucsf.edu (A.R.F.); michael.beattie@ucsf.edu (M.S.B.); jacqueline.bresnahan@ucsf.edu (J.C.B.); 7 Veterans Administration Medical Center, San Francisco, CA 94121, USA

**Keywords:** ultrasound, intraoperative, spinal cord injury, contusion, non-human primate

## Abstract

Background: A spinal cord contusion injury is among the most clinically relevant models for studying pathophysiology and for developing potential therapeutic interventions for spinal cord injuries (SCI). Methods: In this study, we implemented an intra-operative ultrasound (IOU) approach to precisely locate and examine the lesion site at 5 and 10 min post-injury after a cervical hemi-contusion injury in a non-human primate (NHP) model. We assessed acute lesion progression from 5 to 10 min and then compared that to the lesion extent as measured by MRI 3 weeks later. Results: We observed a small increase in the rostrocaudal and mediolateral lesion area (mm^2^) from 5 to 10 min and a further 26% increase in the mediolateral lesion extent when comparing 5 and 10 min to 3 weeks post-injury. Conclusions: By enabling high-resolution ultrasound visualization of the hemicontusion lesion in vivo, this approach can provide critical insights into the early progression of SCI. It can help with further refining this preclinical SCI model and provide significant predictive value for the animals’ recovery post-injury.

## 1. Introduction

In vivo imaging of the spinal cord after traumatic injury is important in clinical decision-making to identify injury severity and determine intervention plans. In clinical care, MRI is the most common imaging modality in the care of SCI patients; however, for large animal models of SCI, access to MRI is costly, requires specialized facilities and staff, and sedation or anesthesia. Intraoperative ultrasound (IOU) is a more accessible and cost-effective technology for characterizing SCI lesions in these models; however, so far, the data on these lesions are limited, and the correlations between IOU, MRI, and injury outcomes are sparse [1]. The few studies that have conducted these observations were in pig thoracic SCI models [2] and not NHP, which makes it challenging for direct clinical translation of the application. We need to understand and quantify how acute IOU and sub-acute MRI measures of injury severity present in the cervical spinal cord to inform cost-effective design of large animal experiments and map the translation to clinical care of people with cervical SCI.

Since the early 1980s, IOU has been used in the clinic to detect the spinal cord and assess the extent of damage due to spinal cord injury (SCI) and other disease processes such as spinal schwannoma tumors [3]. It offers the advantage of providing real-time information without requiring much special patient preparation and can be valuable for examining and assessing intradural lesions, guiding post-injury decompressions, and tumor resections [4,5]. There is evidence of IOU detecting a small-diameter tumor that had remained undetected by other imaging methods, such as MRI [3]. Moreover, other studies have shown that IOU can effectively guide surgeons during procedures involving ventral spinal canal disorders, thus enhancing surgical precision, evaluation of vasculature, and patient outcomes [3,6]. IOU has also been utilized to assess spinal cord blood perfusion and vasculature, providing insights into injury severity and potential prognosis for functional recovery [7].

Based on the wide use and success of IOU in the clinical setting and these applications, we aimed to harness its capabilities to detect, examine, and assess spinal cord lesion progression during the acute phase post-injury. We are using the previously described novel, clinically relevant, spinal cord contusion injury in a non-human primate model [8]. Spinal cord injuries (SCI) can lead to severe motor and sensory impairments. Immediate medical intervention, such as decompression surgery, has been highly correlated with a greater functional recovery prognosis [9,10,11]. This evidence highlights the importance of increasing our understanding (i) of the hyper-acute injury window and (ii) further characterizing the acute SCI pathophysiology. Improved understanding could affect early SCI interventions, thus potentially influencing long-term functional outcomes.

Here, we show how we can utilize the IOU approach for real-time evaluation of the exact location, extent, and progression of the hemi-contusion injury in a non-human primate model. To our knowledge, we are the first to assess how the contusion lesion evolves within those first crucial minutes post-impact. By mapping the dynamic changes that occur in the spinal cord immediately after such contusive trauma, IOU can help us not only further optimize our injury model to improve the reproducibility of experimental studies but also highlight acute aspects of lesion pathophysiology.

## 2. Materials and Methods

### 2.1. Subjects

This study included a total of 21 rhesus macaques (Macaca mulatta) aged 6–15 years (Table 1). All monkeys received a hemi-contusion lesion and intraoperative ultrasound at 5 and 10 min post-impact, and 12 subjects had a 3-week post-injury MRI.

### 2.2. Contusion Lesion

The cervical hemi-contusion injury model was previously described in detail [8]. Briefly, monkeys were sedated and anesthetized, and the C5 dorsal lamina was visualized and removed along with the intervertebral ligamentous tissue and the underlying ligamentum flavum to create an approximately 10 mm wide × 12 mm long laminectomy site. The dura was left intact. The C4 and C6 dorsal spinous processes were clamped to stabilize the vertebral column during the contusion impact. A specially designed positioning device was used to position an impactor rod attached to a precision linear actuator over the spinal cord. The spinal cord midline was determined through a surgical microscope, and the round impactor rod (4 or 5 mm in diameter with a flat plexiglass tip) was positioned with the medial edge of the impactor rod aligned with the spinal cord midline. The impactor position was then adjusted until the medial edge was 0.5–1.2 mm over the midline as previously described. The impactor rod was lowered onto the dural surface to obtain a preload force of 0.5 N (4 mm impactor) or 0.75 N (5 mm impactor) to displace the CSF and trap the cord against the ventral aspect of the vertebral canal. The contusion impact was set to 4 mm at 500 mm/s. The impactor was held in place for 20 msec before being retracted above the cord surface. The resulting impact parameters (displacement, force, and acceleration) were recorded, and impulse and energy were calculated as the integrals of force over time and force over displacement, respectively.

### 2.3. Intraoperative Ultrasound Image Acquisition and Analysis

Before and after impact and prior to the surgeon closing the muscle and incision, we obtained the intraoperative ultrasound images using a Hitachi Arietta 70 (Fujifilm Healthcare). The incision and exposed laminectomy area were flooded with ~25 to 30 cc of sterile saline using a 10 cc syringe. An L53K ultrasound probe (8.5 MHz) was covered with the sterile sleeve, and the surgeon captured mediolateral and rostrocaudal videos and images of the injury site at 5 and 10 min post-impact. Sterile saline was suctioned, the muscle layers and incision were sutured immediately after the acquisition, and the animals recovered. All IOUs were obtained using the same settings on our device (depth, lateral extent, etc.) in order for our comparisons to be valid and compensate for a wide range of animal weights and sizes. Ultrasound videos were recorded in 6 s segments at 30 frames/sec. A minimum of three video segments were recorded at each time point in each of the sagittal and transverse directions.

Ultrasound videos were screened thoroughly to find the best quality video at 5 min and 10 min timestamps for both transverse and sagittal views for each animal. A media player (VLC media player; VideoLAN, Paris, France) was used to convert the selected videos into images using the scene video filter function. A 1:10 recording ratio was set. The image with the epicenter of the lesion was chosen and marked by selecting the image with the lesion of the largest area and brightest intensity. Using ImageJ software (FIJI, v2.14.0/1.54f, NIH, Bethesda, MD, USA), epicenters of lesions on sagittal and transverse views at 5 min and 10 min timestamps were traced. The area of the entire spinal cord was also traced and calculated in pixels. Calibration of the images was performed using the included scale bar to convert pixels to mm^2^. The lesion percentage was calculated (lesion area/whole cord area*100) for the transverse plane.

### 2.4. Magnetic Resonance Imaging Acquisition and Analysis

Animals were sedated with ketamine (Ketaset 10 mg/kg [to effect, range 5–30 mg/kg], intramuscularly (IM); Mylan Institutional LLC, Wilmington, DE, USA) and atropine (0.05 mg/kg intramuscular; Baxter HealthCare Corp, Deerfield, MA, USA), intubated, and maintained using isoflurane (1.5–2.0%; Piramal Critical Care Inc., Bethlehem, PA, USA). Animals were placed in an MRI-compatible stereotaxic device (Model 1430 M; David Kopf Instruments). A 3T Siemens MAGNETOM Skyra scanner using a custom-built four-channel Clamshell MRI coil (Model P-H04LE-030-01295 V01; Rapid MR International, Columbus, OH, USA) was used to acquire magnetic resonance images. Images included high-resolution 3D T2-weighted isotropic scans oriented in the sagittal plane with a slice thickness of 270 μm. MRI images were analyzed by measuring the mediolateral aspects of the spinal cord and lesion, respectively. The spinal cord and lesion areas were measured in mm^2^ and then converted to lesion area % of the intact spinal cord to be comparable with IOUs.

### 2.5. Data Analysis

Data were collected and tabulated. GraphPad Prism software (San Diego, CA, USA) was used for data and statistical analysis. Paired *t*-tests were used to compare changes in lesion area between ultrasound timepoints. Paired *t*-tests were also used to compare differences in lesion area between 10 min ultrasound and 3-week MRI. Statistical differences with *p* < 0.05 were considered significant.

## 3. Results

We used a cervical hemi-contusion injury in the rhesus monkey (n = 21 monkeys were designated for our stem cell graft project in which a hemi-contusion injury and pre-/post-injury ultrasound were performed, and n = 12 were also followed up with post-injury MRI at 3 weeks; Table 1).

**Figure 1:**
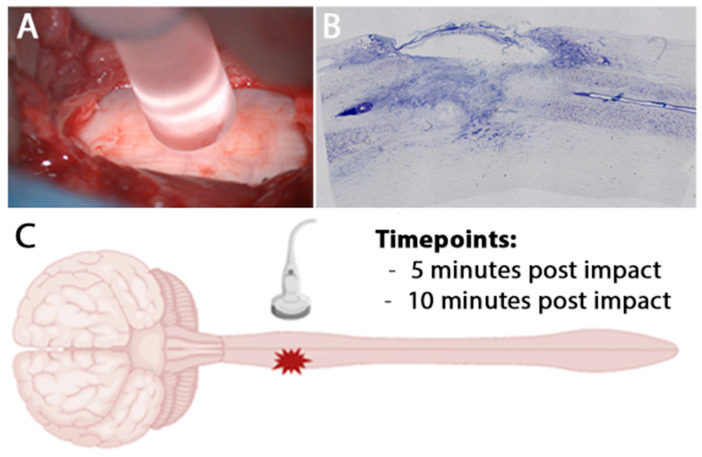
**Acute intraoperative ultrasound of a clinically relevant hemicontusion injury model.** (**A**) Intraoperative photograph showing contusion impactor probe placement directly over the exposed hemi cord following laminectomy. (**B**) Representative NISSL spinal cord section showing hemicontusion morphology and lesion extent. (**C**) Schematic of the experimental paradigm: Intraoperative ultrasound was applied transmurally over the injury site at 5 and 10 min post-injury.

We examine the lesion progression in real time immediately post-impact (5 and 10 min) and at 3 weeks post-injury. As described previously, a hemi-contusion injury was performed by removing the C5 dorsal lamina, securing C4 and C6 spinous processes in clamps, and placing an impactor rod over the exposed dura (Figure 1A) that delivers a controlled impact to one side of the spinal cord (Figure 1B; see methods) (10). Prior to injury, we acquired a baseline intra-operative ultrasound (IOU) of the intact cord transdurally. Less than five minutes post-impact, the rod was removed, the spinous processes unclamped, and the incision area flooded with sterile saline to perform the first IOU at the 5 min point (Figure 1C). The surgeon acquired sagittal and transverse videos and imagery, then waited another 5 min to capture the 10 min post-injury time point. Moreover, a subset of these monkeys (n = 12) received a post-lesion MRI approximately three weeks post-injury.

### 3.1. Intra Operative Ultrasound to Visualize a Hyper Acute Timepoint in a Hemicontusion

**Figure 2:**
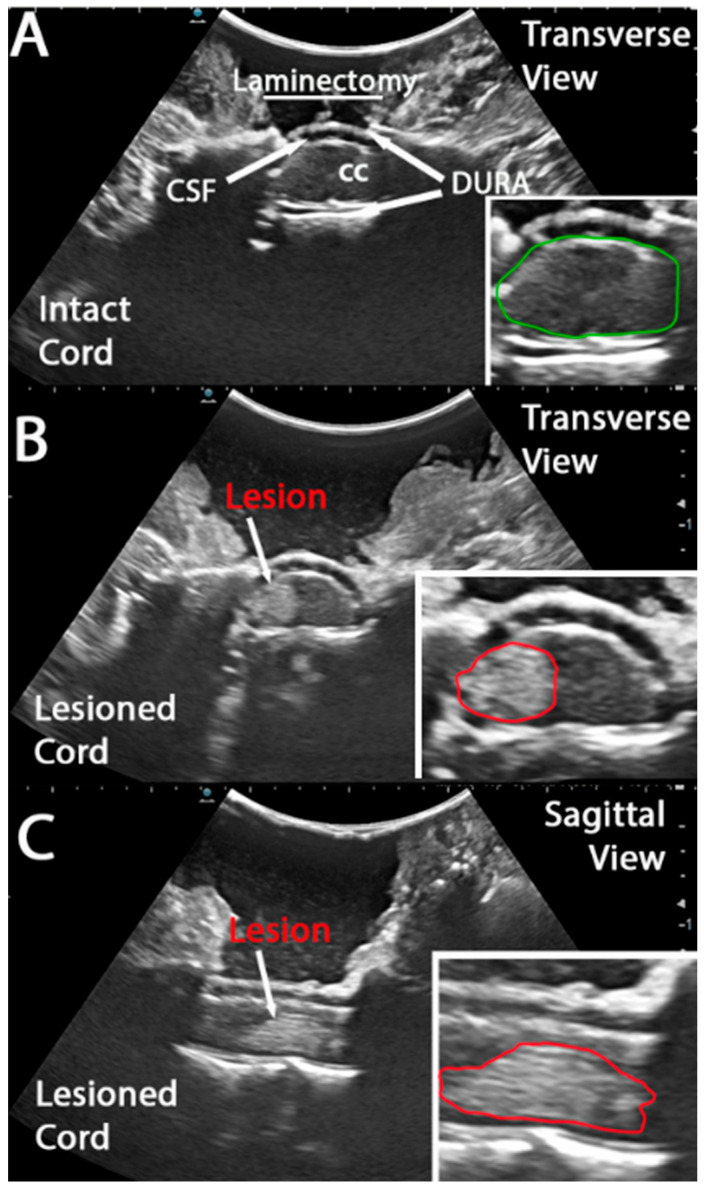
**Ultrasound imaging of intact and lesioned spinal cord in a non-human primate hemicontusion model enables early lesion detection**. (**A**) Coronal ultrasound image of an intact spinal cord following laminectomy. The central canal (marked as CC), dura, and cerebrospinal fluid (marked as CSF) spaces are clearly visualized. Inset: The green outline delineates the intact cord contour. (**B**) Transverse ultrasound image of a lesioned spinal cord. The lesion is marked with a white arrow and red outline (inset). (**C**) Sagittal ultrasound image of the same lesioned spinal cord, with the lesion area visibly disrupting the rostrocaudal continuity of the cord. Similarly, the lesion is marked with a white arrow and red outline (inset).

IOU was performed using a Hitachi Arietta 70 US apparatus, L53K ultrasound probe (8.5 MHz) with a 22 mm field of view. After covering the spinal cord with sterile saline, the probe was manually positioned over the surgical site to capture images and video. As described above, videos and images of the intact spinal cord (Figure 2A) were acquired prior to impact. The intact cord was visualized in the intradural space (Figure 2A and inset; marked with green outline). The contusion device apparatus was then positioned over the cord, impacted, and removed immediately. The surgical site was again flooded with sterile saline for post-impact IOU image acquisition. Impact-derived tissue disruption was clearly visible in both transverse (Figure 1B) and sagittal (Figure 1C) views. Tissue damage was contained within the dura and only on the impacted side of the spinal cord (Figure 2B and insets marked with red outline).

### 3.2. Rostrocaudal and Mediolateral Lesion Extent Increases Significantly Hyper Acutely Post Impact

**Figure 3:**
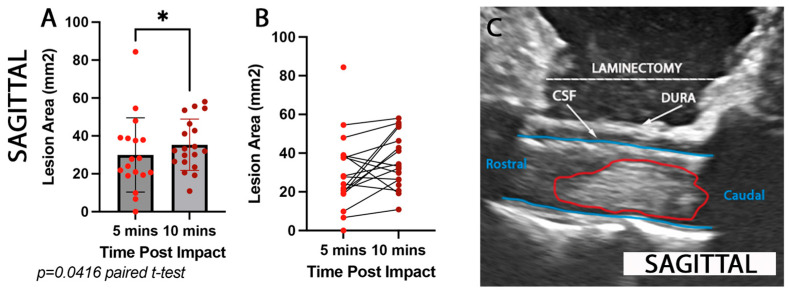
**Comparison of spinal cord rostrocaudal lesion extent detected by ultrasound at 5 and 10 min post-impact**. Quantification of (**A**,**B**) rostrocaudal lesion area (mm^2^) assessed by ultrasound imaging at 5 min (bright red dots) and 10 min (dark red dots) post-injury. The rostrocaudal lesion increased by 5.38 mm^2^ in the first 10 min post injury (* *p* = 0.0416). Intraoperative ultrasound images show an example of (**C**) rostrocaudal (sagittal) lesion epicenter (red outline).

The lesion area (mm^2^) was calculated at 5 and 10 min post-impact at the injury epicenter. We calculated the lesion area using mm^2^. It was not possible to calculate the lesion % of the intact cord in the sagittal plane. An increase of 5.4 mm^2^ was observed in the rostrocaudal (sagittal) progression of the lesion area (t = 2.228, df = 15, paired *t*-test, *p* = 0.0416; Figure 3A–C) from 5 to 10 min after impact. Please note that two animals (Figure 3B) do not have corresponding 10 min IOU measurements due to technical difficulties at the time of surgery.

**Figure 4:**
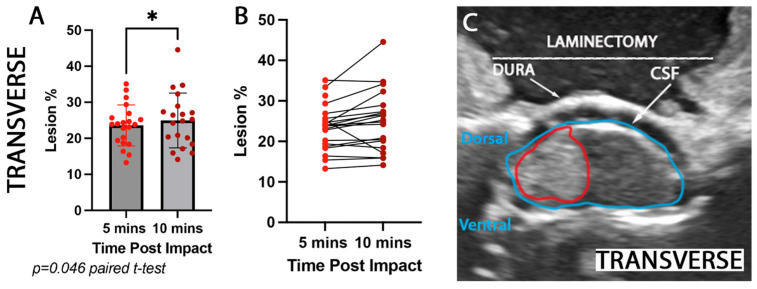
**Comparison of spinal cord lesion percentage of the mediolateral lesion extent detected by ultrasound at 5 and 10 min post-impact**. Quantification of (**A**,**B**) mediolateral lesion area (mm^2^ converted to lesion %—area expressed as percentage of total intact cord area) assessed by ultrasound imaging at 5 min (bright red dots) and 10 min (dark red dots) post-injury. The mediolateral lesion increased by 1.15% in the first 10 min post injury (* *p* = 0.046). Intraoperative ultrasound images show an example of (**C**) rostrocaudal (sagittal) lesion epicenter (red outline), entire cord (blue outline).

Furthermore, we calculated the lesion area for 5 and 10 min post-impact at the injury epicenter using the transverse plane (Figure 4C). In this plane, the calculations were made in mm^2^ and then converted to lesion % (the amount of lesion (red outline) that covers the whole cord (blue outline (Figure 4C)) in order to subsequently compare to MRI measurements. An increase of 1% was observed in the transverse plane of the lesion area (t = 2.126, df = 19, paired *t*-test, *p* = 0.046; Figure 4A,B) from 5 to 10 min after impact.

### 3.3. Mediolateral Lesion Extent Increases Significantly at 3 Weeks Post Impact

**Figure 5:**
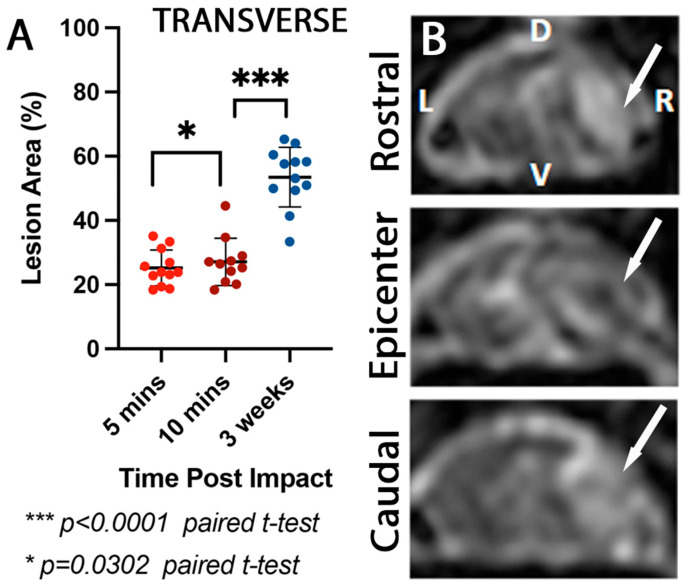
**Comparison of transverse spinal cord lesion size detected by ultrasound at 5 and 10 min post-impact (US) and transverse MRI at 3 weeks post-injury**. (**A**) Quantification of lesion area (expressed as percentage of total intact cord area) assessed by ultrasound imaging at 5 min (US5, orange dots) and 10 min (US10, purple dots) post-injury, compared to lesion area measured by a T2—MRI (blue dots) at 3-week post-injury. Lesion size detected by MRI was significantly larger than that observed by ultrasound at either time point (*** *p* < 0.0001). (**B**) Representative axial MRI images of the spinal cord at rostral, epicenter, and caudal levels relative to the lesion, with arrows indicating areas of hyperintensity consistent with tissue damage. Sagittal MRI image of the spinal cord showing the rostrocaudal extent of the lesion (arrow). All animals showed a significant lesion % increase (47.7 ± 21%) between the immediate IOU and the 3-week T2 view MRI, ranging from 25% to 72%. D: Dorsal, R: Right, L: Left, V: Ventral.

A subset of monkeys received MRIs 3 weeks post-injury (see Table 1). Here, we compared this subset’s 5 and 10 min transverse IOU lesion measurements to examine the lesion progression from a hyperacute timepoint (5–10 min post-lesion) to an acute timepoint (3 weeks post-lesion). Within this subset, an average increase of 1.3% of the lesion area percentage was observed in the hyperacute first 10 min post-lesion (5 vs. 10 min; t = 2.523, df = 10, paired *t*-test, *p* = 0.0302; Figure 4A). This change is similar to the change observed in the entire group as mentioned above (Figure 3A). Moreover, we compared the 10 min IOU with the 3-week post-MRI lesion extent (Figure 5B). We observed an increase in the acute 3-week post-injury lesion area of 26% (10 min vs. 3 weeks; t = 14.51, df = 10, paired *t*-test, *p* < 0.0001; Figure 4A).

## 4. Discussion

The ability to monitor the progression of a clinically relevant spinal cord lesion, such as a hemi-contusion, in real-time can provide a critical step toward improving the accuracy, reproducibility, and translational relevance of large animal preclinical models. In this study, we used rhesus macaques and successfully demonstrated that intraoperative ultrasound (IOU) enables immediate, dynamic, and high-resolution visualization of lesion progression at a hyperacute time point. Our findings indicated that the rostrocaudal lesion area increases 23.4% within the first 10 min post-impact when compared to the initial 5 min timepoint. This result highlights the rapid evolution of SCI pathophysiology of tissue and vasculature damage in the acute phase. Moreover, although there was no evident acute change in the mediolateral extent of the lesion area, a 30% increase was observed at the 3-week time point when the lesion area was measured by MRI and compared to IOU at the 5 min post-injury time point. This result is supported by other MRI studies (on rodents) showing an increase in lesion volume for the first 8 h post-injury [12].

### 4.1. Strengths/Limitations

While intraoperative ultrasound provides a unique and immediate view of the acute spinal cord lesion after impact, the resolution of the images, the precision of handheld probe positioning and orientation, the distortion of images due to bony structures, and the manual image segmentation limit the image quality of IOU. There are new tools, such as 3D ultrasound, that can enable more precise volumetric measures; however, this requires additional fixturing and time to acquire images that may be affected by breathing and other artifacts [6]. In addition, manual segmentation of IOU images to define lesion areas is user-dependent and affected by intra- and inter-rater reliability [2]. In this study, this was mitigated by using a single rater for all segmentation and measurement activities. While image thresholding has been proposed as a more objective method for quantifying intraparenchymal hemorrhage on IOU, these threshold values are highly dependent on image quality. Manual segmentation was implemented for this initial exploration of IOU in a cervical NHP contusion injury model for efficiency and to accommodate intersubject and imaging variability. However, to reduce subjectivity and enable automated image analysis in the future, we will implement standardized image acquisition settings and image thresholding. Also, it has to be noted that in our experimental setting, using a non-human primate contusion model, these IOUs have been extremely helpful in providing critical insights for immediate post-injury animal recovery. We were able to alert the veterinary staff regarding the animals where the IOUs showed that the injury had crossed the midline, and that some extra care might be necessary. Moreover, given that this is a non-human primate study, the absence of controls to assess procedural influences on the lesion (such as potential IOU evoked heat) is a limitation of this study that needs to be considered as a potential constraint for the generalizability and interpretation of our results; however, there is little to no evidence in the literature showing IOU detrimental effects. Finally, for similar reasons, we did not have the opportunity to examine IOUs at the 3-week point [13]. We understand that the visualization of lesion progression could have been slightly altered by the difference in imaging methodology.

### 4.2. Comparison with Other Contusion Injury Models Using US to Characterize Lesions or Preinjury Morphology

There have been very few observations of acute intraoperative ultrasound of spinal cord contusion injuries. Our work provides the first observations of these ultra-acute lesions in a cervical hemicontusion model in a non-human primate. Our IOU observations of increasing rostral/caudal spread of the lesion after impact using sagittal images of the lesion epicenter provide a novel observation of acute injury progression in a large animal SCI contusion model. Prior work by Kwon et al. mapping the hourly progression of contusion injuries in the rodent spinal cord after thoracic contusion was only able to characterize the transverse spread of the lesion as images were acquired axially [14]. In the short (10 min) post-injury observation time, we did not observe significant changes in axial lesion area. In particular, the contralateral spread of the unilateral lesion observed immediately after injury (5 min) was consistent with observations at 10 min post-injury and 3-week post-injury MRI. This is important as contralateral spread of a cervical unilateral injury has significant implications in the severity of functional deficits, the animal care protocols and approvals, and the intensity of postoperative care.

### 4.3. Accessibility of IOU vs. MRI and Its Potential Utility in Preclinical Studies

Intraoperative ultrasound is relatively more affordable than MRI (clinical US systems are in the $100K USD versus >$2M USD for MRI) and provides the advantage of real-time information for the surgeon. In preclinical large animal models, MRI access can be prohibitively expensive or not even possible due to the specialized facilities and staff required for MRI. While there continue to be challenges regarding the resolution of intraoperative spinal cord ultrasound, new technology developments, including higher resolution probes [15] and 3D volumetric scanning [16], have the potential to address these concerns.

## 5. Conclusions

In this study, we successfully demonstrated that intraoperative ultrasound (IOU) is a valuable tool for the real-time, high-resolution visualization of spinal cord lesion progression in a clinically relevant non-human primate model of cervical hemi-contusion. Our findings reveal a rapid and significant expansion of the lesion in both rostrocaudal and mediolateral dimensions within the first 10 min post-injury, highlighting the dynamic nature of hyper-acute spinal cord injury (SCI) pathophysiology. A further substantial increase in lesion extent was observed at 3 weeks post-injury when compared to acute IOU measurements, confirming the progression of secondary injury.

This approach offers critical insights into the early evolution of SCI, which can help improve the reproducibility of experimental injury models, provide predictive value for animal recovery, and guide immediate post-operative care.

## Figures and Tables

**Table 1:** Animal SCI contusion details.

Subject	Age	Impactor Head Size	Max Cord Displacement (mm)	Impactor Head Placement Beyond Cord Midline	Impact Force	MRI
1	15 yrs	5 mm	4.01	1 mm	13.7 N	YES
2	8 yrs	5 mm	3.73	1 mm	27.02 N	YES
3	11 yrs	5 mm	3.97	1 mm	19.9 N	YES
4	7 yrs	5 mm	3.75	1 mm	29 N	YES
5	11 yrs	5 mm	3.70	1 mm	26.5 N	YES
6	6 yrs	4 mm	3.86	0.7 mm	26.9 N	YES
7	9 yrs	5 mm	3.67	1 mm	23.4 N	YES
8	12.9 yrs	5 mm	3.92	1 mm	9.9 N	NO
9	8 yrs	5 mm	3.64	1 mm	24.5 N	NO
10	6 yrs	5 mm	3.99	1 mm	5.6 N	NO
11	7.5 yrs	5 mm	3.96	1 mm	20.8 N	YES
12	9 yrs	5 mm	3.70	0.7 mm	18.7 N	NO
13	6.5 yrs	5 mm	4.04	1.1 mm	13.4 N	YES
14	8 yrs	5 mm	3.89	0.5 mm	17.6 N	NO
15	8 yrs	5 mm	3.74	1 mm	14.4 N	YES
16	7 yrs	5 mm	3.75	1 mm	23.2 N	NO
17	8 yrs	5 mm	3.99	1 mm	23.1 N	YES
18	7.5 yrs	5 mm	3.96	0.5 mm	10.3 N	NO
19	8 yrs	4 mm	4.11	0.55 mm	15.3 N	NO
20	7.5 yrs	5 mm	4.01	1 mm	12.1 N	YES
21	8 yrs	5 mm	3.85	1 mm	24.3 N	NO

## Data Availability

The raw data supporting the conclusions of this article will be made available by the authors on request.

## Abbreviations

The following abbreviations are used in this manuscript:

IOU	Intraoperative ultrasound
NHP	Non-human primate
SCI	Spinal cord injury
